# Editorial: Evolving economies in sports: management practices and market impacts

**DOI:** 10.3389/fspor.2026.1871944

**Published:** 2026-05-19

**Authors:** Philip Kang, Sehwan Kim, María Huertas González Serrano

**Affiliations:** 1 Department of Kinesiology, Augusta University, Augusta, GA, United States; 2 C.H. Sandage School of Business, Graceland University, Lamoni, IA, United States; 3 Department of Physical Education and Sport, Universitat de València, València, Spain

**Keywords:** analytics, digitalization, economics, governance, legitimacy

For much of sport management scholarship, economic analysis has been anchored to familiar concerns such as ticket sales, sponsorship revenue, media rights, labor costs, and competitive balance. Those concerns remain important, but they are no longer sufficient for understanding how value is created and sustained in contemporary sport. Evolving economies in sport should not be understood simply as a matter of growth, decline, or commercial expansion. The phrase captures deeper shifts in the mechanisms through which value is produced, measured, justified, and redistributed across sport systems.

The nine articles in this special issue collectively demonstrate that economic change in sport is inseparable from management practice. Markets are not static environments that organizations merely enter and exploit. They are actively reorganized through decisions about models, rules, infrastructures, identities, and legitimacy. Taken together, the contributions show that contemporary sport economies are being reshaped not only by financial pressures but also by changes in how organizations forecast performance (1–3), assign value to assets, maintain stakeholder trust (4–6), manage institutional disruption, and respond to broader technological and policy environments (7–9).

## Competitive analytics, valuation, and strategic decision systems

The first set of contributions demonstrates that evolving sport economies are increasingly shaped by analytical systems designed to improve competitive decisions. Weirich et al. (3) revisit one of the best-known forecasting approaches in American football and show that machine learning models can outperform the Pythagorean expectation framework in predicting NFL wins. Their use of SHAP-based interpretation also reinforces an important point for sport managers: model performance alone is not enough. Analytical systems become more useful when decision makers can understand which performance indicators are driving the output and how those signals can be translated into organizational action.

Hadley et al. (1) make a parallel intervention in the domain of draft pick valuation. Their study challenges the continued reliance on traditional trade value charts by showing that those charts may misprice picks and understate the asymmetrical cost of trading up. In doing so, the article reframes draft strategy as a problem of economic judgment rather than simple competitive intuition. Pradhan and Leshchinskii (2) similarly examine whether NBA teams that exceed the soft salary cap through luxury-tax spending obtain performance gains large enough to justify the additional financial burden. Although the context differs, the underlying question is similar: under what conditions does greater spending or more aggressive resource deployment produce strategically meaningful returns?

## Legitimacy, trust, and the social foundations of economic value

A second group of articles shows that fan relationships, institutional trust, and public legitimacy are not external to the sport economy. They are among their core foundations. Hernández-Hernández et al. (4) examine how football fans in Colombia evaluate clubs' efforts to balance economic and social outcomes. Their findings suggest that supporters do not assess organizational performance solely through financial indicators or sporting success. Rather, club value is filtered through a broader understanding that includes social contribution, responsibility, and relational meaning. This is a useful corrective to narrow economic framings that assume stakeholders care only about competitive results and financial stability.

Reichel et al. (6) reinforce that point through their study of trust and mistrust in Russian football fan relationship management. By centering governance arrangements, including Fan ID, the authors show that institutional trust is not a background condition but an actively contested dimension of sport markets. Whether fans view governing bodies and club authorities as trustworthy has direct implications for engagement, compliance, and the long-term durability of the relationship between organizations and their publics. Trust, in other words, is not merely symbolic, it has economic consequences.

Karlsson (5) further extends this line of thinking through the Falsterbo Horse Show case, where sponsorship becomes contested through moral and nationalist discourse. The article demonstrates that the economic value of sponsorship is never purely contractual. Commercial partnerships must survive public interpretation. When sponsors become associated with moral compromise, political controversy, or symbolic threat, the market value of the partnership becomes unstable. Across these three papers, the common lesson is clear: economic value in sport is always socially mediated. Clubs, leagues, and events do not simply generate value; they must also secure the legitimacy that allows that value to endure.

## Digital infrastructure, institutional shock, and adjacent market strategy

Finally, the third cluster of articles highlights the extent to which sport economies are now shaped by systems that extend beyond the traditional boundaries of teams and leagues. Zhang et al. (8) show that digital payments significantly influence sports lottery consumption and that this relationship is strengthened by the surrounding business environment. Their study suggests that consumer behavior in sport-related markets cannot be understood purely through individual demand. Participation is also structured by transaction systems, payment infrastructure, and the institutional quality of the market environment.

Zhang et al. (9) push this infrastructural perspective further by examining the relationship between the digital economy and the green transformation of the sports industry. Their findings indicate that digital development can facilitate greener industry outcomes, particularly where the marketization of data factors is more advanced. The article therefore expands the meaning of economic change in sport by showing that industry development is increasingly tied to digital governance, sustainability imperatives, and broader policy conditions.

Webster and Carr (7), in a very different context, argue that the post-House era of college athletics may compel universities to rethink how sport revenue is generated and protected. Their conceptual analysis points to real estate development and adjacent commercial strategies as possible responses to a more volatile and labor-intensive college sport environment. The value of the article lies not only in the specific proposal, but also in the broader reframing it offers. College athletics can no longer be understood through legacy revenue models alone. Institutional disruption now forces managers to consider alternative asset configurations and hybrid business strategies.

## Critical issues and future research directions

The articles in this special issue point to several priorities for future research. First, the field needs stronger frameworks for distinguishing technical performance from decision usefulness. Better prediction, more refined valuation, and statistically significant economic effects are important, but they do not automatically translate into adoption or organizational impact. Future work should examine how sport organizations incorporate analytical tools into real decision processes, how internal politics shape their uptake, and when technically superior evidence is ignored because it threatens established routines or power structures. The field gains little from increasingly sophisticated models if it does not also understand the organizational conditions under which those models matter (1–3).

Second, the issue reveals the need for stronger theorization of hybrid value in sport. Several articles show that economic, social, moral, and environmental outcomes are not separate domains that can be analyzed in isolation. Clubs are evaluated not only on financial or sporting performance, but also on their perceived social role. Sponsorships are judged through moral and political narratives. Industry development is increasingly assessed through sustainability criteria. These are not secondary considerations to be added after the economic analysis is complete. They are central to how organizations justify their actions and maintain support. Future research should therefore move beyond simplistic distinctions between economic and non-economic outcomes and develop more integrated accounts of how multiple forms of value are negotiated simultaneously (4, 5, 9).

Third, the special issue underscores the importance of institutional unevenness. Economic change in sport does not unfold uniformly across organizations, sectors, or regions. The Chinese studies explicitly identify regional heterogeneity. The post-House college athletics analysis implies that some institutions will be far better positioned than others to pursue new revenue strategies. The Russian and Swedish cases demonstrate that governance and sponsorship are interpreted through distinct cultural and political conditions. Future studies should ask not only whether a strategy works, but for whom, under what circumstances, and with what distributional consequences. Sport economies do not evolve evenly, and sport management research should stop writing as if they do (5–9).

Lastly, this issue suggests that sport economies would benefit from more dialogue across subfields. Competitive analytics, payroll strategy, fan trust, sponsorship legitimacy, digital payments, sustainability, and revenue adaptation are often studied as separate problems. In practice, they are deeply interconnected. A roster-spending decision can alter public perceptions of organizational responsibility. A sponsorship controversy can affect the symbolic and commercial value of a sport property. A digital-payment ecosystem can reshape participation in regulated sport products and influence broader public-welfare concerns. A stronger field will study these domains as interconnected processes rather than isolated topics. Doing so would generate a more realistic and more useful understanding of how value circulates through contemporary sport systems (2, 5, 6, 8).

## Conclusion

The nine articles in this special issue make a compelling case that sport economies are evolving not merely because there is more money in sport, but because the rules, infrastructures, metrics, and legitimacy conditions that govern value creation are being rewritten. Some contributions show how competitive decision-making is becoming more data-driven, valuation-sensitive, and resource-conscious. Others demonstrate that fan trust, public meaning, and moral contestation shape the viability of clubs, leagues, and events.

Collectively, these studies push sport management scholarship toward a broader and more realistic account of economic change. They suggest that the field can no longer afford to treat economics as a narrow question of revenue maximization, labor cost, or market exchange alone. Contemporary sport economies are co-produced by analytics, governance, technology, discourse, and institutional context. The contribution of this special issue, therefore, is not simply that it documents change. It shows that the management of sport now requires a wider economic imagination, one capable of recognizing that value must be forecast, priced, justified, protected, and repeatedly reassembled under changing conditions.
